# An OxPLORE Initiative Evaluating Children’s Surgery Resources Worldwide: A Cross-sectional Implementation of the OReCS Document

**DOI:** 10.1007/s00268-021-06377-w

**Published:** 2021-11-30

**Authors:** Krupa Ravi, Annabel Killen, Angus Alexander, Frances Bell-Davies, James Biganiro Sebintu, Aurelia Brazeal, Jean Marie Vianney Butoyi, Fabio Edgardo Diaz, Romeo Drabile, Marvin Fanny, Lucila Fernie, Shannon Gunawardana, Emma Hartley, Yolisa N. Hawu, Holly Hendron, Stephanie Alcine Joseph, Ananda Lamahewage, Ruwantha Mahagedera, Emery Manirambona, Benjamin Kitambala Morisho, Patrick Muchunu, Alliance Niyukuri, Edmond Ntaganda, Francisco Orliacq, Josefina Orliacq, Adili Wobenjo, Pablo Young, Kokila Lakhoo, Kathryn Ford

**Affiliations:** 1 Medical Sciences Division, University of Oxford, Oxford, UK; 2 Department of Paediatric Surgery, The Children’s Hospital at Westmead, Westmead, Australia; 3 College of Medicine and Health Sciences, University of Rwanda, Kigali, Rwanda; 4 Kenyatta University, Nairobi County, Kenya; 5 Saint Therese Hospital, Bujumbura, Burundi; 6 Hospital Británico de Buenos Aires, Buenos Aires, Argentina; 7 Department of Paediatric Surgery, Sefako Makgatho Health Sciences University, Tshwane, Gauteng Province South Africa; 8 Seychelles Hospital, Victoria, Seychelles; 9 King’s College Hospital, London, UK; 10 University of Sydney, The Children’s Hospital at Westmead, Westmead, Australia; 11 Central Coast Local Health District, New South Wales, Australia; 12 Department of Paediatric Surgery, Sefako Makgatho Health Sciences University, Pretoria North, Gauteng Province South Africa; 13 Royal Sussex County Hospital, University Hospitals Sussex NHS Foundation Trust, Brighton, UK; 14 Ministry of Health, Victoria, Seychelles; 15 Lady Ridgeway Hospital, Colombo, Sri Lanka; 16 Mutoyi Hospital, Mutoyi, Burundi; 17 Department of General Surgery, Kiambu County Hospital, Kiambu, Kenya; 18 Mercy Surgeons-Burundi, Research Department, Bujumbura, Burundi; 19 Hope Africa University Frank-Ogden Medical School, Bujumbura, Burundi; 20 Pediatric Surgery Service, Centre Hospitalier Universitaire de Kigali, Kigali City, Rwanda; 21 Pontificia Universidad Católica Argentina, Buenos Aires, Argentina; 22 Department of Paediatric Surgery, University of Oxford and Oxford University Hospitals, Oxford, UK; 23 Department of Population, Policy and Practice, Institute of Child Health, University College London, London, UK; 24 Oxford University Global Surgery Group, Nuffield Department of Surgical Sciences, John Radcliffe Hospital, University of Oxford, Room 6607, Level 6, Headington, Oxford, Ox3 9du UK

## Abstract

**Background:**

The Global Initiative for Children's Surgery (GICS) group produced the Optimal Resources for Children’s Surgery (OReCS) document in 2019, listing standards of children’s surgical care by level of healthcare facilities within low resource settings. We have previously created and piloted an audit tool based on the OReCS criteria in a high-income setting. In this study, we aimed to validate its use in identifying gaps in children’s surgery provision worldwide.

**Methods:**

Our OReCS audit tool was implemented in 10 hospitals providing children’s surgery across eight countries. Collaborators were recruited via the Oxford Paediatrics Linking Our Research with Electives (OxPLORE) international network of medical students and trainees. The audit tool measured a hospital’s current capacity for children’s surgery. Data were analysed firstly to express the percentage of ‘essential’ criteria met for each specialty. Secondly, the ‘OxPLORE method’ was used to allocate each hospital specialty a level based on procedures performed and resources available. A User Evaluation Tool (UET) was developed to obtain feedback on the ease of use of the tool.

**Results:**

The percentage of essential criteria met within each category varied widely between hospitals. The level given to hospitals for subspecialties based on OReCS criteria often did not reflect their self-defined level. The UET indicated the audit tool was practicable across multiple settings.

**Conclusions:**

We recommend the use of the OReCS criteria to identify areas for local hospital improvement and inform national children’s surgical plans. We have made informed suggestions to increase usability of the OReCS audit tool.

## Introduction

An estimated 1.7 billion children and adolescents across the world lack access to surgical care [1]. This burden is predominantly carried by low- and middle-income countries (LMICs), with the 2015 Lancet Commission on Global Surgery highlighting a significant deficit in children’s surgical provision compared to demand in these countries [2]. The Global Initiative for Children's Surgery (GICS), a consortium of healthcare professionals caring for children with surgical needs founded in 2016 [3], used LMIC-led working groups to develop guidelines aimed at addressing the inequity in children’s surgical provision [4]. These guidelines, mapped out in the Optimal Resources for Children's Surgery (OReCS) document, provide a list of procedures that the health system 'should be capable of performing safely', and standards for optimal resources, divided into 'training & staffing', 'physical resources' and 'quality and safety' (Online Resource 1) [5].

Existing capacity assessment tools for children’s surgery have largely been developed by groups based in high-income countries (HICs) and are not attuned to the specific needs of LMIC surgical providers. PediPIPES, developed by Surgeons OverSeas Assessment of Surgical Need (SOSAS), is an exception to this, yet lacks the breadth of enquiry into personnel availability and procedures being performed that the OReCS document incorporates [6]. There is a need for a tool which is designed for LMIC children’s surgical providers and able to objectively measure key aspects of surgical capacity and performance in these settings [7].

We have previously piloted our OReCS audit tool in a HIC, with results used by one level 2 hospital to guide commissioning [8]. Here, we aim to investigate the effectiveness of the OReCS audit tool in assessing children’s surgical provision across all resource settings. Additionally, we sought user feedback to explore practicality of tool use within participating healthcare centres.

### Methods

Oxford Paediatrics Linking Our Research with Electives (OxPLORE) is an international research network that encourages collaboration between medical students and trainee doctors on elective placements to contribute to projects investigating relevant children’s surgery issues [9]. It is coordinated by a team of medical students from different healthcare settings and supervised by a paediatric surgery trainee (KF) and consultant paediatric surgeon (KL).

Recruitment of collaborators (medical students or trainee doctors from the UK or LMICs) for this study was initiated through the OxPLORE network in April 2019, with data collection finishing in October 2020. Collaborators were required to identify a consultant paediatric surgeon supervisor at their centre and meet ethical approval or audit registration criteria according to local protocols. Visiting students were required to partner with a local medical student or junior doctor.

The OReCS audit tool was developed (Online Resource 2) and amended according to user feedback following the HIC pilot study [8]. Electronic questions were based on the OReCS document and the WHO Tool for Situational Analysis to Assess Emergency and Essential Surgical Care [5, 10], covering classification of participating centres, access to basic resources and equipment, infrastructure, children’s population served, physician and non-physician workforce and children’s surgical procedures performed according to speciality. The OReCS document defines standards for these categories at each level of facility: health centre (HC), first (1st) level, second (2nd) level, third (3rd) level and National Children’s Hospital (NCH). These criteria were translated into dichotomous survey questions allowing stepwise assessment of surgical provision at each level.

Participants were advised to contact children’s surgery departments, consult hospital managerial staff and search through hospital notes or websites to find answers to the audit questions. The data collected reflected the centre’s current capacity to perform selected procedures and current availability of resources including personnel.

Data were analysed centrally (KR, AK). Any missing data were clarified with collaborators and their supervisors. Data analysis utilised two methods. The ‘essential criteria method’ marks the percentage of essential criteria met by each healthcare centre. Essential criteria are defined as the ‘minimal acceptable level of children’s surgical care’ expected at even the most resource-limited settings in the OReCS document and include standards expected at health centre and first level hospitals [5]. For each category, the number of HC and 1st level criteria met by a centre was divided by the total number of HC and 1st level criteria in that category. This fraction was converted to a percentage.

The ‘OxPLORE method’ gives healthcare centres a score based on the proportion of OReCS criteria concerning human resources or childhood surgical procedures (classified by subspecialty or surgical condition) met. For each category, the number of HC criteria met by a centre was divided by the total number of HC criteria. This was then repeated individually for 1st, 2nd, 3rd and NCH level criteria for the same category. The sum of the fractions was calculated and rounded to the nearest integer. This integer corresponded with an overall Disease Control Priorities 3 (DCP3) level, with 1 equating to HC level and 5 equating to NCH level (Online Resource 3).

An electronic user evaluation tool (UET) was sent to each collaborator after completion of data collection (Online Resource 4). Data were gathered on ease of use of the OReCS-based audit tool, methods and barriers concerning data collection and interpretation of questions.

## Results

**Fig. 1:**
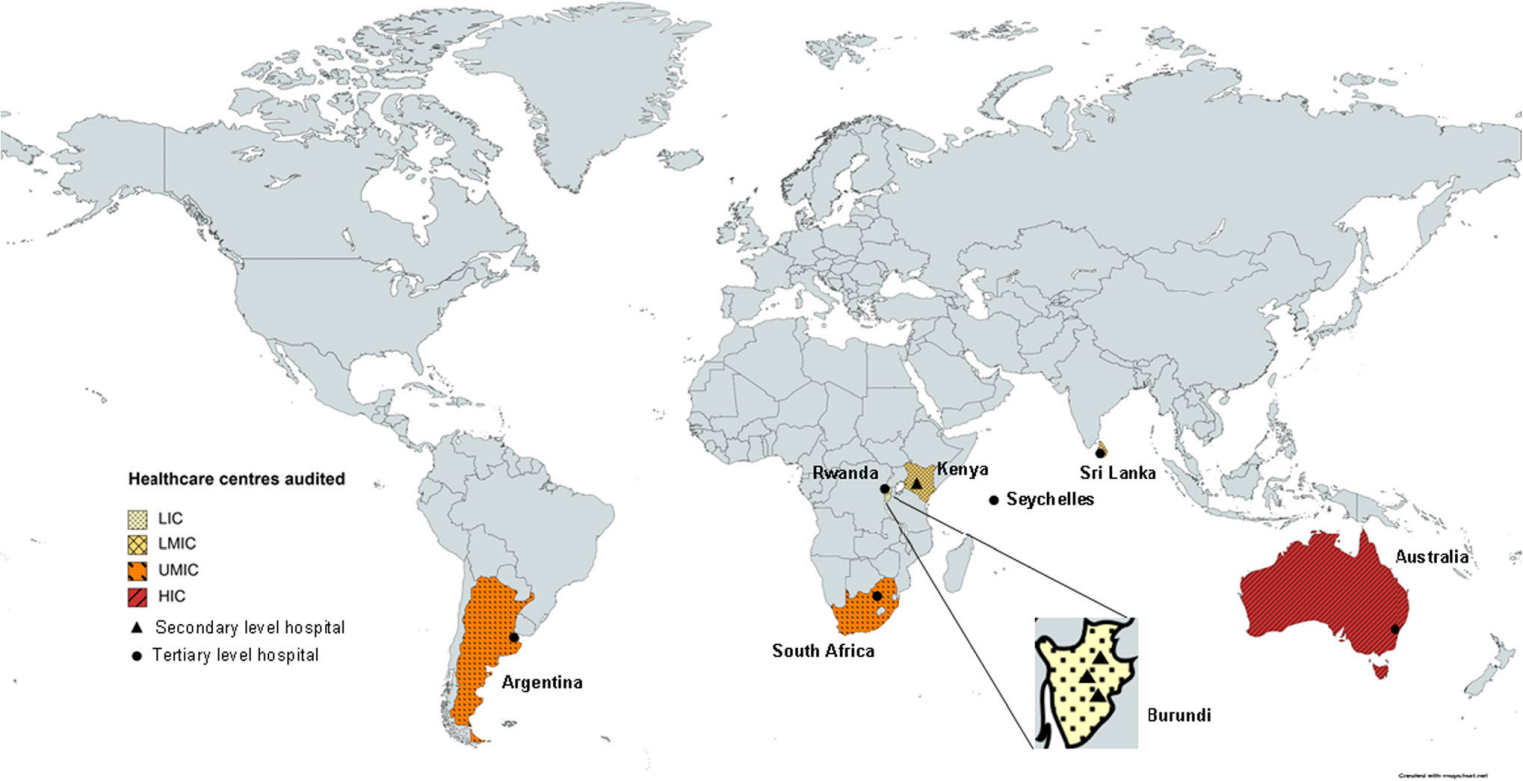
Map showing the geographical location of the 10 collaborating centres; the economic status for each country is per World Bank definition. Figure generated using MapChart and Microsoft Publisher

Ten centres providing children’s surgery participated in the study (Fig. 1). Two centres were in HICs, and the remaining eight in LMICs. Public, private and mission hospitals were included. Three centres served a paediatric population nationwide, and the remaining seven treated regionally (Table 1).

**Table 1:** Hospital characteristics including the presence or absence of paediatric and neonatal intensive care units

Country	World Bank Income Classification		Classification	Type of healthcare facility	Size of paediatric population served	PICU	NICU
Burundi	LIC	Hospital 1	Secondary	Mission	Regional	N	N
		Hospital 2	Secondary	Private	Regional	N	N
		Hospital 3	Secondary	Mission	Regional	N	N
Rwanda	LIC		Tertiary	Public	Nationwide	Y	N
Kenya	LMIC		Secondary	Public	Regional	N	N
Sri Lanka	LMIC		Tertiary	Public	Nationwide	Y	Y
South Africa	UMIC		Tertiary	Public	Regional	Y	Y
Argentina	UMIC		Tertiary	Private	Regional	Y	Y
Seychelles	HIC		Tertiary	Public	Nationwide	N	Y
Australia	HIC		Tertiary	Public	Regional	Y	Y

LIC, low-income country; LMIC, lower-middle-income country; UMIC, upper-middle-income country; HIC, high-income country; PICU, paediatric intensive care unit; NICU, neonatal intensive care units

### ‘Essential criteria method’

No hospital met all essential criteria in every domain (Table 2). Seven out of the ten centres did not meet 100% essential criteria for HR non-physicians. The absent job types varied between centres. Three centres did not have trained birth attendants, and all three Burundi hospitals did not have physical and occupational therapists. Six centres did not meet all essential criteria for HR physicians, with clinical officers and non-physician surgical providers most commonly missing. The absence of one or both was independent of the size of centre or income level of its country of origin.

**Table 2:** Percentage of essential criteria met for each category and hospital

Country	Burundi			Rwanda	Kenya	Sri Lanka	South Africa	Argentina	Seychelles	Australia
	Hospital 1	Hospital 2	Hospital 3							
Self-defined level	Secondary	Secondary	Secondary	Tertiary	Secondary	Tertiary	Tertiary	Tertiary	Tertiary	Tertiary
Infrastructure	100%	67%	100%	100%	100%	100%	100%	100%	100%	100%
HR Non-physicians	80%	60%	90%	70%	100%	90%	100%	100%	90%	90%
HR Physicians	50%	50%	100%	50%	100%	100%	100%	0%	50%	75%
Anaesthesia	100%	50%	100%	100%	100%	100%	100%	100%	100%	100%
Cardiac surgery	50%	0%	50%	100%	0%	100%	75%	100%	75%	100%
Critical care	100%	38%	100%	100%	100%	100%	100%	100%	86%	100%
General surgery	100%	63%	100%	100%	63%	100%	100%	100%	100%	100%
Neurosurgery	67%	0%	100%	100%	33%	33%	100%	100%	67%	100%
Ophthalmology	80%	20%	100%	100%	100%	100%	100%	100%	100%	100%
Maxillofacial surgery	100%	100%	100%	100%	100%	100%	100%	100%	100%	100%
Orthopaedic surgery	100%	75%	75%	100%	100%	100%	100%	100%	100%	100%
Otolaryngology	100%	0%	100%	100%	86%	100%	100%	100%	100%	100%
Plastic surgery	100%	60%	100%	100%	100%	100%	100%	100%	100%	100%
Urology	86%	43%	100%	100%	86%	100%	100%	100%	100%	100%
Trauma—resus	33%	33%	100%	100%	67%	100%	100%	100%	100%	100%
Trauma—injuries	75%	75%	100%	100%	75%	100%	100%	100%	100%	100%
Trauma- fractures	100%	100%	100%	100%	100%	100%	100%	100%	100%	100%
Trauma—burns	100%	100%	100%	100%	100%	100%	100%	100%	100%	100%
Congenital anomalies	20%	20%	80%	70%	50%	100%	90%	100%	30%	100%
Infections	78%	56%	100%	100%	100%	100%	100%	100%	100%	100%
Tumours	67%	0%	100%	100%	33%	100%	100%	100%	100%	100%
Other treatments	80%	40%	100%	80%	80%	100%	100%	80%	80%	80%

Seven centres did not meet all essential criteria for congenital anomalies. There was a deficit both in screening for anomalies, such as neural tube defects, and management of anomalies found. Six centres did not provide medical treatment with prostaglandin for patent ductus arteriosus (PDA) dependent disease.

There were also seven centres which didn’t meet 100% of essential criteria for ‘other treatments’. Five of these (all but those in Burundi) fall below 100% due to not performing male circumcision using Plastibell.

### ‘OxPLORE method’

Analysis by the ‘OxPLORE method’ also highlighted HR non-physicians and physicians, congenital anomalies and ‘other treatments’ as areas where some centres are not performing procedures concurrent with the level of hospital based on the OReCS document (Table 3). It also indicated that many centres are exceeding performance expectations in anaesthesia, general surgery and management of burns. A description of pertinent findings for each centre is in Online Resource 5.

**Table 3:** Level given to each category in each hospital using the OxPLORE method

Country	Burundi			Rwanda	Kenya	Sri Lanka	South Africa	Argentina	Seychelles	Australia
	Hospital 1	Hospital 2	Hospital 3							
Self-defined level	Secondary	Secondary	Secondary	Tertiary	Secondary	Tertiary	Tertiary	Tertiary	Tertiary	Tertiary
Infrastructure	Intermediate	Intermediate	Intermediate	Intermediate	Intermediate	Complex	Complex	Complex	Intermediate	Complex
HR Non-physicians	1st	1st	1st	2nd	2nd	3rd	3rd	3rd	2nd	NCH
HR Physicians	HC	HC	1st	2nd	2nd	3rd	2nd	NCH	1st	2nd
Anaesthesia	2nd	HC	NCH	NCH	NCH	NCH	NCH	NCH	NCH	NCH
Cardiac surgery	HC	< HC	1st	3rd	< HC	NCH	1st	NCH	2nd	NCH
Critical care	1st	1st	NCH	3rd	3rd	NCH	NCH	NCH	3rd	NCH
General surgery	NCH	HC	NCH	NCH	1st	NCH	NCH	NCH	NCH	NCH
Neurosurgery	2nd	< HC	3rd	NCH	HC	1st	NCH	NCH	2nd	NCH
Ophthalmology	3rd	HC	2nd	3rd	3rd	NCH	NCH	NCH	3rd	3rd
Maxillofacial surgery	1st	1st	2nd	2nd	1st	NCH	1st	NCH	2nd	NCH
Orthopaedic surgery	2nd	2nd	NCH	NCH	3rd	NCH	NCH	NCH	3rd	NCH
Otolaryngology	2nd	< HC	2nd	3rd	2nd	NCH	NCH	NCH	3rd	NCH
Plastic surgery	3^rd^	1st	NCH	NCH	2nd	NCH	3rd	NCH	2nd	NCH
Urology	2nd	HC	2nd	3rd	1st	NCH	NCH	NCH	2nd	NCH
Trauma—resus	HC	HC	1st	1st	1st	NCH	NCH	NCH	1st	NCH
Trauma—injuries	2nd	1st	3rd	2nd	1st	3rd	NCH	NCH	2nd	NCH
Trauma- fractures	2nd	1st	NCH	NCH	2nd	2nd	NCH	NCH	NCH	NCH
Trauma—burns	NCH	NCH	NCH	NCH	NCH	NCH	NCH	NCH	NCH	NCH
Congenital anomalies	HC	HC	1st	2nd	HC	3rd	3rd	NCH	1st	NCH
Infections	2nd	1st	NCH	3rd	2nd	NCH	NCH	NCH	NCH	NCH
Tumours	2nd	< HC	2nd	NCH	HC	3rd	NCH	NCH	2nd	NCH
Other treatments	1st	HC	3rd	2nd	1st	3rd	NCH	3rd	2nd	3rd

### User evaluation tool (UET)

**Fig.2:**
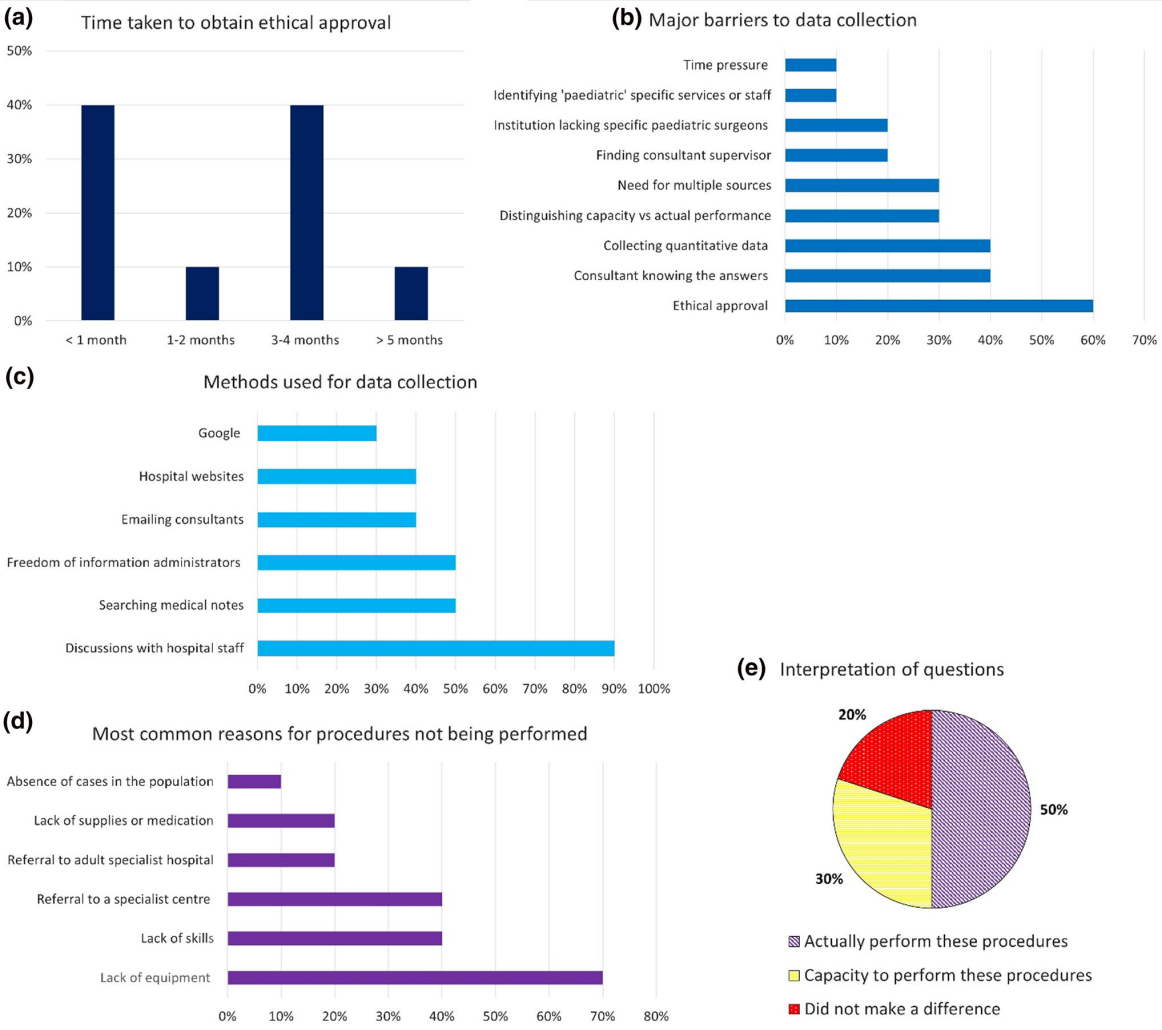
Graphical representation of the results of the user evaluation tool showing **a** time taken to obtain ethical approval; **b** prevalence of different barriers to data collection; **c** data collection methods; **d** reasons for procedures not being performed; **e** how the data collectors interpreted the questions proposed in the OReCS audit proforma concerning whether surgical procedures are performed. Figure generated using Microsoft Excel

We received completed feedback from six medical students, two junior doctors and two consultants. None of those responding to the survey had previously heard of the ORECS audit tool, although three of the students or juniors reported their senior supervisors had. The time taken to get the ethical approval required before starting showed variation (Fig. 2a), acting as a major barrier to collecting data for 60% (6/10) of individuals (Fig. 2b). There was a dichotomy in time required for data collection, with the process taking between one day and one week for some individuals, and over a month for others. Many data collectors utilised multiple methods, with discussion with hospital staff the most frequently cited (Fig. 2c).

Most data collectors found lack of equipment the most common reason for procedures not being performed (Fig. 2d). The survey also gave insight into some of the limitations of the audit tool, showing variation in how the questions regarding procedures were interpreted (Fig. 2e). Certain terms were deemed difficult to interpret whilst some procedures, such as treatment of leprosy, were inappropriate to certain centres. Furthermore, the criteria within some categories were too prescriptive, for example, the requirement for circumcision using Plastibell within ‘other treatments’ disadvantages centres who perform circumcision using other techniques. Job titles such as non-physician surgical provider (an essential role for a 1st level hospital) are not universal, leading to hospitals being marked down despite potentially employing staff with a similar role.

**Fig. 3:**
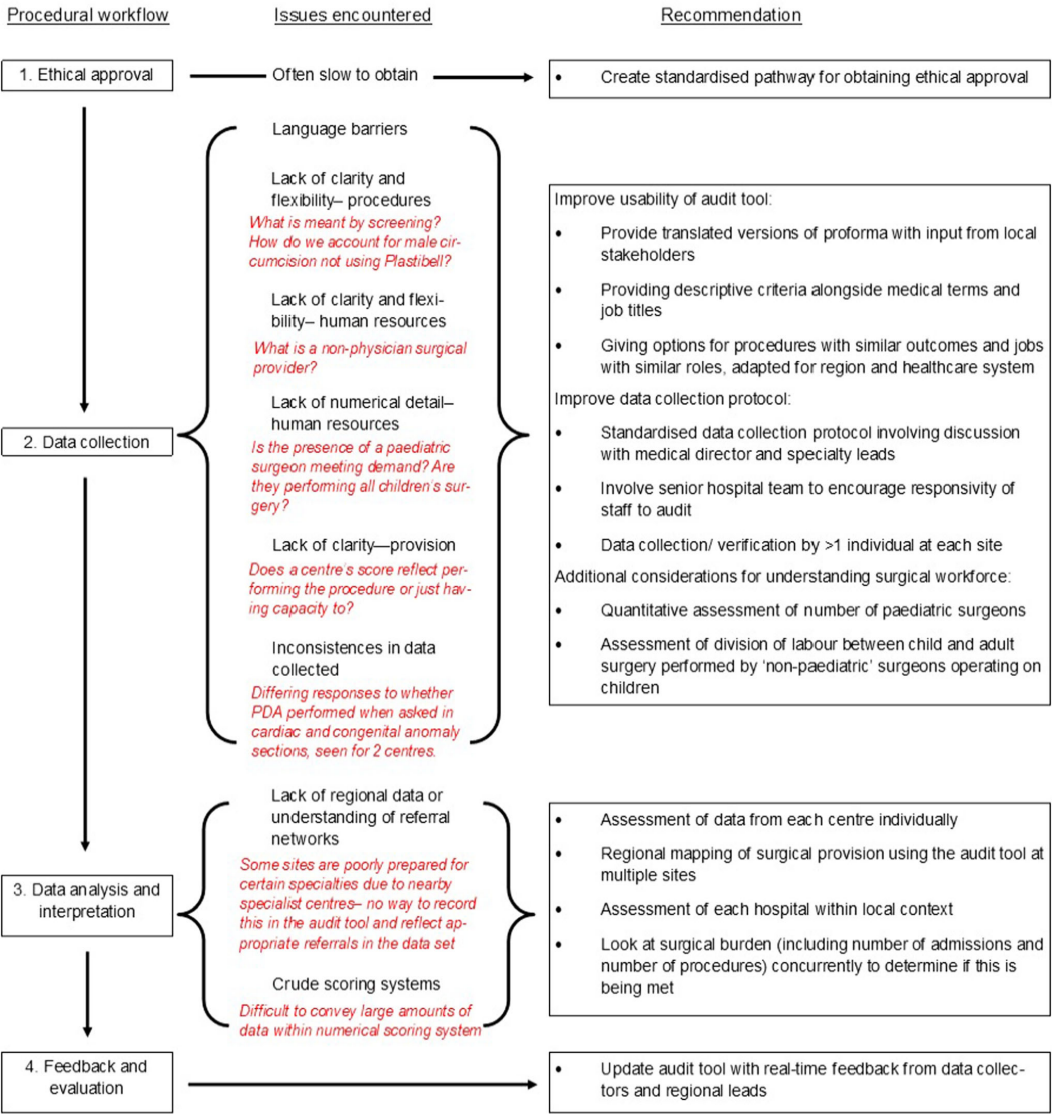
Flowchart showing issues encountered at each stage of the audit process as indicated by the UET, discussion with data collectors and in data analysis, alongside examples of such issues (red and italicised) and recommendations for future usage of the audit tool. Figure generated using Microsoft Publisher

Specific questions (such as ‘number of children at this facility requiring surgery’) did not give a clear time frame and were difficult to quantify. Data collectors also reported uncertainty over whether congenital screening questions related to newborn exams or prenatal ultrasounds. Despite these difficulties, the OReCS audit tool was deemed easy to use, with all respondents scoring ease of use as at least six out of ten, and 60% (6/10) scoring it nine or ten (with ten indicating most ease). 80% (8/10) respondents believed doctors in the hospital they were auditing were keen to promote research, although 30% (3/10) experienced some problems in finding a consultant supervisor. A series of recommendations for future use of the OReCS tool based on issues identified from the UET have been suggested (Fig. 3).

## Discussion

### Key findings

Our results show the OReCS tool can be used in multiple settings to evaluate children’s surgical provision. It effectively highlighted where centres were not meeting 100% essential criteria, with the ‘OxPLORE method’ of analysis revealing specialities where a hospital is under- or over-performing compared to expected. Further analysis of the score breakdown showed where more complex speciality procedures were being performed, despite procedures deemed simpler not occurring.

The UET suggested users found the tool straightforward, although implementation of certain changes (such as refining questions so they are country, region, and children’s surgical provider-relevant) could improve usability. Clear descriptions of roles required at each level would also allow the tool to highlight areas requiring greater personnel training and recruitment more accurately.

### Strengths

This multi-centre analysis of children’s surgical provision used an objective audit tool, which could be translated to facilitate use in other countries. The healthcare centres audited were diverse, with representation from each of the World Bank economic status categories. Data were gathered contemporaneously, reflecting the capacity for children’s surgery of a centre at that time.

Data analysis was performed individually for each centre using two methods. The ‘essential criteria method’ was an indicator of where basic hospital procedures were lacking. This provides an overview of overall hospital performance and could be particularly useful for planning development on a regional or national level. The ‘OxPLORE method’ allowed more in-depth analysis, such as might be necessary for planning resource allocation within a hospital. For example, showing where more basic procedures are not occurring within an otherwise well-developed subspecialty could represent resource-efficient ways to increase capacity for children’s surgery.

Through the UET, we obtained subjective feedback on tool use in different settings. This has led to recommendations (Fig. 3) which consider feasibility of performing this audit in LMICs, where the need for such data is greatest, with a focus on increasing ease of use alongside reliability of results.

A final strength of the study is that it has facilitated the growth of the OxPLORE collaborative international network. This network has the potential to form the basis of ‘regional research hubs’—an additional aim set out by the OReCS document [5].

### Limitations

The main limitation of our study is the absence of quantitative data for reporting surgical capacity. Without this, we are unable to comment on whether the population’s needs are being met. A complete understanding of this issue would also involve knowledge of a region’s burden of disease, and thus for the OReCS tool to be fully useful, population-level and epidemiological research is first required. This in turn requires a certain level of resource, training and time, which may not be available in all settings.

Our data rely on individual reporting and so may have introduced reporter bias. This could result in under- or over-reporting of resources and may differ between institutions. We do not anticipate this to influence the reliability of our results for two reasons. Firstly, our primary aim was to implement the OReCS audit tool in multiple settings and report on its usability. Secondly, we did not aim to conduct a comparison between the children’s surgery providers.

### Implications and recommendations

This is the first translation and implementation of the OReCS document; a tool unique in delivering detailed criteria focussed on the specific surgical needs of children in LMICs. We have suggested several recommendations to mitigate issues identified at each stage of the implementation process (Fig. 3). A widespread rollout of the audit tool would benefit from regional leads who are responsible for local recruitment of institutions and data collectors. These individuals, working as part of regional research hubs, could adapt the standardised pathway for obtaining ethical approval and the language used in the OReCS audit tool to suit the local setting. Furthermore, use of the OReCS tool in serial audit cycles (with regular use of a user evaluation tool to allow adjustments in response to local feedback) could allow evaluation and refinement of policy changes and interventions.

We suggest the OReCS audit tool should be updated to include quantitative assessment of surgical burden and current provision, enabling the results to be used to guide allocation of resources and training, and to inform policy. At a national level, these data could inform children’s surgical planning alongside national surgical, obstetric and anaesthesia plans.

## Conclusion

Worldwide, millions of children lack essential surgical care. Objective assessment of the performance of hospitals in their local context is fundamental to addressing this unmet need. Through the established OxPLORE research network, we have created and tested a tool which benchmarks the provision of children’s surgery based on international consensus. The OReCS audit tool successfully identified gaps in children’s surgery provision across multiple settings. Furthermore, our research has enabled us to make recommendations to increase usability of the tool, increasing its utility in guiding resource allocation worldwide.
